# Immobilized and Free Cells of *Geotrichum candidum* for Asymmetric Reduction of Ketones: Stability and Recyclability

**DOI:** 10.3390/molecules23092144

**Published:** 2018-08-27

**Authors:** Hui Liu, Fayene Zeferino Ribeiro de Souza, Lan Liu, Bi-Shuang Chen

**Affiliations:** 1 School of Marine Sciences, Sun Yat-Sen University, Guangzhou 510275, China; liuh229@mail2.sysu.edu.cn; 2 South China Sea Bio-Resource Exploitation and Utilization Collaborative Innovation Center, Sun Yat-Sen University, Guangzhou 510275, China; 3 Departamento de Química, Faculdade de Ciências, UNESP, Bauru 17033-360, Brazil; faylittlefay@yahoo.com.br

**Keywords:** immobilization, free cells, asymmetric reduction, stability, reusability

## Abstract

Marine-derived fungus *Geotrichum candidum* AS 2.361 was previously reported by our group as an active strain for the enantioselective reduction of ketones. Although some other *Geotrichum* strains were also found from the terrestrial sources, information on their stability and reusability is scarce. Herein, the stabilities—in terms of pH tolerance, thermostability, and storage stability, and reusability—of *G. candidum* AS 2.361 were described for the asymmetric reduction of a series of aromatic ketones. Two differently immobilized cells (agar immobilization and calcium alginate immobilization) as well as free cells were prepared. For three substrates (1-(3-bromophenyl) ethan-1-one (**1b**), 1-(2-chlorophenyl) ethan-1-one (**1d**), and acetophenone (**1g**)) immobilized cells on agar showed a great improvement in the bioreduction activities compared to the free cells, increasing yields up to 97% with *ee* values of 99%. Cells immobilized on agar/calcium alginate could maintain more than 90% of the original activities within the assayed pH ranges of 3.5–11, while free cells were highly sensitive to alkaline and acidic conditions. Concerning thermostability, immobilized cells on agar kept 99% of their original activities after incubation at 60 °C for 1 h, while almost no activity was detected for the free cells under the same condition. Immobilized cells were stable at 4 °C for 80 days without any activity loss, while free cells started to decrease the activity after storage at 4 °C for six days. The immobilized cells retained almost 99% activity after four reuse cycles, while free cells lost almost all the activities at on the third cycle.

## 1. Introduction

Enantiomerically pure alcohols are important building blocks that are often found as common structural motifs in the production of bioactive compounds [1]. For example, 2-bromo-1-phenylethanol is a versatile intermediate for the synthesis of fluoxetine, tomoxetine, and nisoxetine, which are used for *anti*-depressants and α- or β-adrenergic drugs [2,3]. With the increasing demand for the chiral alcohols, their synthesis has continuously attracted great research interest. Among the methods used for the production of chiral alcohols, asymmetric reduction of prochiral carbonyl compounds is generally considered to be a reliable, scalable, and straightforward route to enantiomerically pure alcohols. Compared to traditional chemical methods, biocatalysis offers significant advantages, such as remarkable chemo-, regio-, and stereoselectivity; environmentally benign processes; and energy-efficient operations [4,5]. Whole-cell biocatalysts are more attractive rather than isolated enzymes used in the asymmetric reduction of ketones for the production of chiral alcohols because the whole-cell system can generate sufficient necessary cofactors for the reduction by cellular metabolism [6]. Indeed, whole-cells of a number of microorganisms have been used as biocatalysts for enantioselective preparation of chiral alcohols [7,8,9,10]. Unfortunately, to the best of our knowledge, most of the known microorganisms have not been applied for industrial preparation of chiral alcohols, owing in large part to the relatively low catalytic activity and stereoselectivity of these microorganisms.

Novel and robust microorganisms with perfect catalytic activity and stereoselectivity in the fields of biocatalytic reduction are in great demand. Future success can be achieved from the exploitation of marine microorganisms’ enzymatic potential [11,12,13,14,15,16]. Marine microorganisms are thoroughly adapted to surviving and growing under extreme environments, resulting in their production of novel enzymes with optimal biochemical properties at harsh conditions (extreme temperature, pressure, pH, or organic solvent) [17,18]. Such habitat-related characteristics make marine microorganisms ideal biocatalysts of asymmetric bioreduction for the production of the desired enantiomerically pure alcohols [19,20,21].

In our previous work [19,20,21], 13 marine-derived fungus strains were characterized as active strains for the catalytic activity in the enantioselective reduction of 13 aromatic ketones of different compound classes. Excellent-to-good yields and enantioselectivities were achieved with these marine-derived fungi, such as *Rhodotorula mucilaginosa*, *R. rubra*, *Penicillium citrinum*, *Aspergillus sydowii*, *A. sclerotiorum*, and *G. candidum* AS 2.361. Notably, strain *G. candidum* AS 2.361 showed promising bioreduction activity; this strain has been found by other groups from terrestrial sources [22,23,24], but it was first discovered from a marine source.

However, information on its stability and reusability is scarce. Moreover, the stability (including pH tolerance, solvent tolerance, thermostability, and storability) and reusability are highly important characteristics of a biocatalyst from the viewpoint of process economics [25,26]. These observations have motivated us to investigate the stability and reusability of strain *G. candidum* AS 2.361 for the asymmetric reduction of ketones.

In the case of soluble cells, they tend to form aggregates with altered properties and that may alter the results of the activity and stability studies and suggests that the use of the free cells may not be very adequate. The stability of whole-cells can be strongly enhanced by immobilization *via* entrapment in polymers such as agar and alginate gels [27].

The term ‘immobilization’ designates the application of physical or chemical methods (e.g., physical adsorption, ion-coagulation, crosslinking, and entrapment) to make the enzyme into insoluble derivatives catalyzing biochemical reactions. Cell entrapment is one of the simplest methods and most widely used for cell immobilization, where the cells are enclosed in agar and calcium alginate beads which are attractive for a number of preparations in biotechnology, biomedicine, cosmetic and edible fields. However, the use of immobilized cells is in many instances associated with a decrease in activity produced by slight distortions in the cells’ structure or diffusional limitations; although in some cases an increase in cells activity is achieved [28].

Therefore, it is meaningful to include using free cells for the asymmetric reduction of ketones. Here, we report the results of a comparative study on the asymmetric reduction of a variety of ketones by immobilized and free cells of *G. candidum* AS 2.361.

## 2. Results and Discussion

### 2.1. Characterization of Immobilized Cells

#### 2.1.1. FTIR Spectroscopy

**Figure 1:**
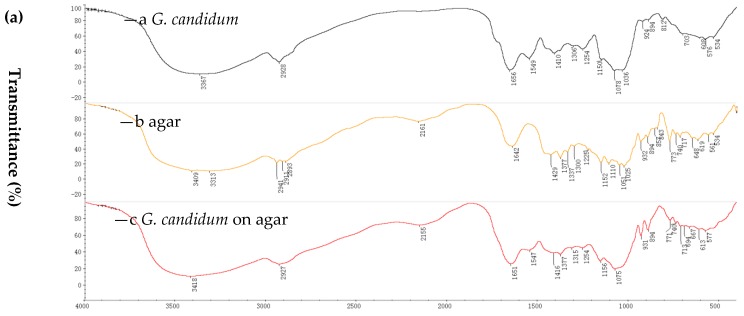

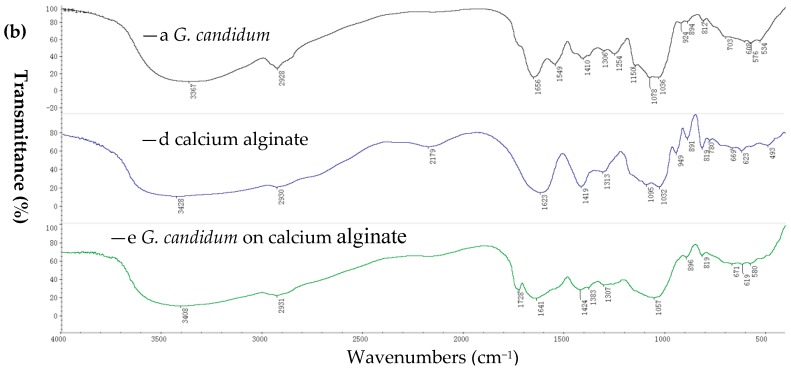
Fourier transform infrared (FTIR) for immobilization of *G. candidum* AS 2.361 on agar (**a**), on calcium alginate (**b**): —a *G. candidum* AS 2.361; —b agar; —c *G. candidum* AS 2.361 on agar; —d calcium alginate; —e *G. candidum* AS 2.361 on calcium alginate.

The characterization of immobilized cells and non-immobilized cells was performed by Fourier transform infrared spectroscopy (FTIR) and scanning electron microscopy (SEM). FTIR spectra were used to elucidate the functional groups of the immobilized cell matrices [29]. The immobilization of marine-derived *G. candidum* AS 2.361 was affected with two support matrices, namely, agar and calcium alginate. The choice of the two supports was based on our previous report and data about the immobilization of the fungi *R. mucilaginosa* [21]. The immobilization on PVA-alginate and chitosan were also prepared, however, resulting in significant decrease in activity produced by slight distortions in the cells’ structure or diffusional limitations. Thus, the immobilization cells on PVA-alginate and chitosan were not used for further studies. Spectra were recorded in the range of 4000–400 cm^−1^ using a Hitachi 270-50 IR spectrophotometer with a KBr disc. FTIR spectra showing the results obtained for free cells (of *G. candidum* AS 2.361), native agar and immobilized cells on agar are summarized in Figure 1a ((a) is for *G. candidum* AS 2.361; (b) is for native agar; (c) is for immobilized *G. candidum* AS 2.361 on agar). In particular, peaks at 3313 cm^−1^ (O–H stretching), 2911 cm^−1^ (C–H stretching), and 1152 cm^−1^ (C–O–C stretching) as shown in Figure 1a (b) are the characteristic peaks of native agar. Comparing the spectra of native agar (Figure 1a (b)) and immobilized cells on agar (Figure 1a (c)), it is clear that the position and intensity of the characteristic peaks of native agar are significantly changed or completely lost, clearly confirming that the immobilization has occurred. In addition, the presence of a new peak at 1651 cm^−1^ (Figure 1a (c)) provides further support for the occurrence of the immobilization.

FTIR spectra showing free cells (of *G. candidum* AS 2.361), native calcium alginate and immobilized cells on calcium alginate are summarized in Figure 1b ((a) is for *G. candidum* AS 2.361; (b) is for calcium alginate; (c) is for immobilized *G. candidum* AS 2.361 on calcium alginate). In particular, peaks at 3428 cm^−1^ (O–H stretching), 1032 cm^−1^ (C–O–C stretching), 1623 cm^−1^ and 1419 cm^−1^ (–COO asymmetric and symmetric stretching) as shown in Figure 1b (b) are the characteristic peaks of native calcium alginate. Comparing the spectra of native calcium alginate (Figure 1b (b)) and immobilized cells on calcium alginate (Figure 1b (c)), it is clear that the position and intensity of the characteristic peaks of native calcium alginate are significantly changed or totally lost, clearly confirming that the immobilization has occurred. In addition, the presence of a new peak at 1728 cm^−1^ (Figure 1b (c)) provides further support for the occurrence of the immobilization 

#### 2.1.2. SEM Spectroscopy

**Figure 2:**
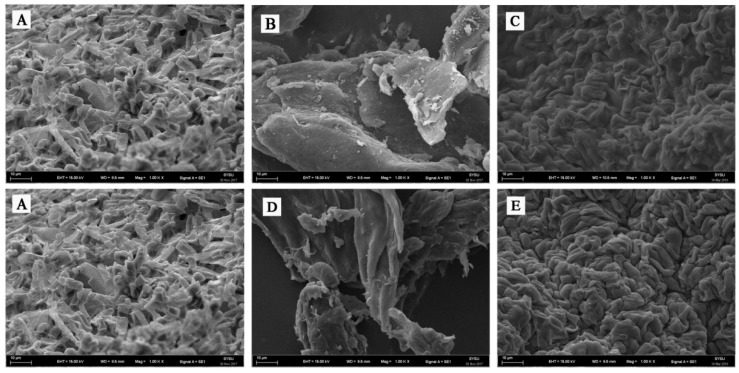
Scanning electron micrographs of agar and calcium alginate beads containing *G. candidum* AS 2.361 cells. (**A**) free cells of *G. candidum* AS 2.361; (**B**) agar; (**C**) *G. candidum* AS 2.361 cells immobilized on agar; (**D**) calcium alginate; (**E**) *G. candidum* AS 2.361 cells immobilized on calcium alginate.

The immobilization reaction was further confirmed by SEM imaging. The SEM images of agar and calcium alginate immobilization are presented in Figure 2. The SEM images of free *G. candidum* AS 2.361 cells, native agar and immobilized *G. candidum* AS 2.361 cells on agar are shown as Figure 2A–C, respectively. Figure 1B shows the smooth surface of native agar. Figure 2C clearly showed that *G. candidum* AS 2.361 cells were captured in the agar microspheres, cross-linking with agar using distilled water. SEM studies further proved that *G. candidum* AS 2.361 cells were well immobilized on the agar surface, most likely owing to the flocculating ability as well as cross-linking.

The SEM images of free *G. candidum* AS 2.361 cells, native calcium alginate and immobilized *G. candidum* AS 2.361 cells on calcium alginate are presented in Figure 2A,D,E, respectively. Figure 2D shows the smooth surface of calcium alginate. Figure 2E clearly shows that *G. candidum* AS 2.361 cells were captured in the calcium alginate microspheres, cross-linking with calcium alginate via CaCl_2_. SEM studies further revealed a good immobilization of *G. candidum* AS 2.361 cells on the surface of calcium alginate, which is most likely due to the calcium alginate’s high mechanical strength and cross-linking ability. Admittedly, as an immobilization matrix, the gelling properties of an alginate are absolutely essential and are highly dependent on its monomeric composition and sequential arrangement [30]. Our experiments suggested that the formation of microspheres of calcium alginate for capture of *G. candidum* AS 2.361 cells required 4% CaCl_2_ and that complex interactions were occurring between *G. candidum* AS 2.361 cells and calcium alginate [31].

### 2.2. Biocatalytic Activity of Free and Immobilized G. candidum AS 2.361 Cells

The stability of biocatalysts can be significantly enhanced by the simple and easy immobilization technology. Moreover, the immobilized cells can be filtered off easily, offering simple operation. On the other hand, the immobilization process makes the reaction conditions of immobilized cells different from those of the free cells, which can clearly affect enzyme activity and/or make the process no longer viable on an industrial scale. Here, before starting to investigate the stability and recyclability of the immobilized *G. candidum* AS 2.361 cells in the enantioselective reduction of prochiral ketones, we first should make sure that the immobilization is not masking enzyme deactivation. To compare the biocatalytic activity of free and immobilized *G. candidum* AS 2.361 cells for the asymmetric reduction of ketones, both free cells and immobilized cells (on agar and calcium alginate) were used as catalysts for the reactions. Free cells were obtained as described previously [19]. Immobilized cells were obtained according to the protocols described in the Experimental Section 3.2. The reaction mixture contained 3 g of free resting cells or 4.5 g of immobilized cells, 0.5 g of glucose in 10 mL Na_2_HPO_4_-KH_2_PO_4_ buffer (100 mM, pH 7.0), with the substrates of the final concentration of 10 mM. The solution was shaken at 25 °C for 24 h. The biocatalytic activity results in terms of yields and *ee* values are summarized in Table 1.

The data presented in Table 1 clearly indicate that both free *G. candidum* AS 2.361 cells and immobilized *G. candidum* AS 2.361 cells (including immobilized cells on agar and calcium alginate) smoothly reduced the aromatic ketones **1a**–**1j** into the corresponding enantiomerically enriched alcohols. Gratifyingly, no significant difference in activity in terms of the yields and enantioselectivity of the desired chiral alcohols was found between free cells and immobilized cells. Notably, for substrates 1-(3-bromophenyl) ethan-1-one (**1b**), 1-(2-chlorophenyl) ethan-1-one (**1d**) and acetophenone (**1g**), immobilized cells on agar showed a great improvement in the bioreduction activities. Specifically, free *G. candidum* AS 2.361 cells converted substrates **1b**, **1d**, and **1g** into the corresponding enantiomerically enriched alcohols in 44, 81, and 63% yields with *ee* values of 58, 90, and 87%, respectively, while immobilized *G. candidum* AS 2.361 cells on agar converted substrates **1b**, **1d**, and **1g** into the corresponding enantiomerically enriched alcohols in 65, 97, and 86% yields with *ee* values of 99, 99, and 99%, respectively. This finding may be attributed to the fact that the immobilized cells (on agar/calcium alginate) can facilitate the stability of the microbial system and maintain a relatively higher level of ketone reductase production compared to the free cells. Moreover, the agar and calcium alginate in beads have many porous structures, thus allowing the substrates more access to the ketone reductase and accelerating the reduction of the ketones **1b**, **1d**, and **1g** under these conditions. More interestingly, in the case of 2-chloro-1-phenylethan-1-one (**1i**), the reduction was greatly enhanced by the immobilized cells both on agar and calcium alginate. The reaction with the immobilized cells resulted in a total inversion of the configuration obtained from the free cells, yielding the (*R*)-alcohol [(*R*)-**2i**] instead of the (*S*)-alcohol [(*S*)-**2i**]. Although in both cases the enantioselectivity of **2i** was not unsatisfactory, the inversion of the configuration of the (*S*)-alcohol [(*S*)-**2i**] (produced by the free cells) to the (*R*)-alcohol [(*R*)-**2i**] (produced by the immobilized cells) showed strong influence of the immobilization depending on the type of substrates and/or support materials used. The immobilization process may have brought about some changes in the physical properties, which favoured the action of (*R*)-selective carbonyl reductases rather than (*S*)-selective enzymes. Another possibility may be that the immobilization has activated another dehydrogenase which is different from the enzymes catalyzing the reduction in free cells [32]. However, a definite explanation about the inversion of configuration could still not be confirmed in the present study, and this issue is currently being investigated in our laboratory. Overall, agar and calcium alginate are excellent support matrices for the immobilization of *G. candidum* AS 2.361 cells, with beneficial influence on the enzyme activity. In order to prove the usefulness and importance of applied procedures, the reaction was up-scaled to 1000 mL using **1a** as represented substrate and immobilized cells on agar as represented biocatalysts under the same reaction conditions (pH 7.0, 25 °C, 24 h). Gratifyingly, in the preparative-scale reaction immobilization cells with agar catalyzed quantitatively substrate **1a** to the desired product (*S*)-**2a**. The obtained results highlighted the potential industrial use of the applied procedure.

### 2.3. Stability

#### 2.3.1. pH Tolerance

**Figure 3:**
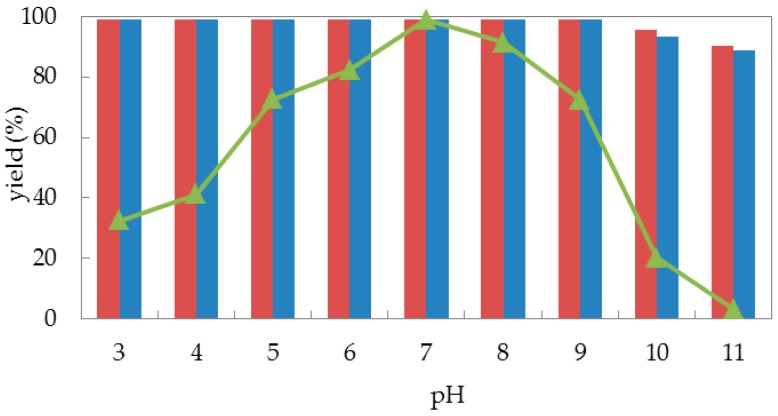
pH profile for free cells (represented by the triangles), immobilized cells on agar (represented by the red striped bar) and calcium alginate (represented by the blue striped bar) of *G. candidum* AS 2.361 catalyzing the reduction of 1-(2-bromophenyl) ethanone (**1a**).

Encouraged by the acceptable biocatalytic activities obtained from both free cells and immobilized cells of *G. candidum* AS 2.361, we then turned our attention to its stabilities. Generally, buffer pH can affect the stability, and/or the activity/selectivity of an enzymatic reaction, that is, pH tolerance is a key factor for the stability of a biocatalyst. Fundamentally, the immobilization process could strongly enhance the stability of an enzyme. Thus, we are interested in the pH effect on the bioreduction activity of *G. candidum* AS 2.361 cells in the forms of both free cells and immobilized cells. Here, reactions were all performed in 50-mL screw-capped plastic vials to prevent evaporation of ketones/alcohols. The reaction mixture contained 3 g of free resting cells or 4.5 g of immobilized cells, 0.5 g of glucose in 10 mL given buffer (100 mM) with given given pH values between 3.5–11.0 (citrate buffer of pH 3.5 and 4.5; phosphate buffer of pH 5.5, 6.5, and 7.5; Tris buffer of pH 8.5, 9.0, 10.0, and 11.0), with the substrates of the final concentration of 10 mM. The solution was shaken at 25 °C for 24 h. As shown in Figure 3, there was no significant difference in the effect of pH on the enzymatic activity of the cells immobilized on agar or calcium alginate (the red striped bar and the blue striped bar). When immobilized cells (on agar and calcium alginate) were tested, both quantitatively catalyzed the reduction of 1-(2-bromophenyl) ethan-1-one (**1a**) to alcohol (*S*)-**2a** (yield >99%) within the assayed pH ranges of 3.5–9.0. When the buffer pH increased from 9.0 to 11.0, both immobilized cells catalyzed slightly less substrates **1a** into the desired alcohols (*S*)-**2a** (yield 90%), corresponding to approximately 90% of the original activity at pH 3.5–9.0. By contrast, the free cells were highly sensitive to alkaline and acidic conditions. As illustrated in Figure 3, the catalytic activities of free cells were strongly affected by the use of different buffers with different pH values. The biocatalytic activities slowly increased with the pH increasing from 3.5 to 7.0, and neutral conditions (pH 7.0) represented the optimal pH for substrate **1a**, with the product yield of 99%. When the pH values continuously increased from 7.0 to 11.0, the catalytic activity of free cells dramatically decreased, and almost no activities were detected at pH 11.0. Remarkably, the reaction product *ee* remained above 99% in the measured pH ranges (not shown in Figure 3). This strongly emphasizes that pH had a weaker effect on the immobilized cells than on the free cells because the immobilization of the cells can protect them from harsh environmental conditions, such as highly acidic or highly alkaline environments [37]. Indeed, immobilization can protect enzyme to some extent from solvent denaturation and eventually from inactivation at extreme conditions, by creating a stable microenvironment [38]. In summary, free cells of *G. candidum* AS 2.361 were sensitive to buffer pH, with dramatic biocatalytic activity decreases in acidic and alkaline conditions, while immobilized cells of *G. candidum* AS 2.361 exhibited excellent resistance in the measured pH ranges (3.5–11), with no significant changes of biocatalytic activities.

#### 2.3.2. Thermostability

**Figure 4:**
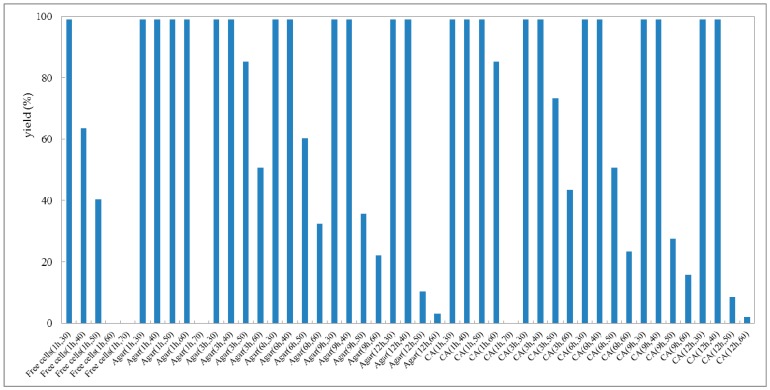
Thermostability of free cells and immobilized cells on agar and calcium alginate (CA) of *G. candidum* AS 2.361 catalyzing the reduction of 1-(2-bromophenyl) ethanone (**1a**). Cells were incubated at different temperatures for different times before the substrate was added to initiate the reactions. Agar (1 h, 30) represents immobilized cells on the agar were incubated at 30 °C for 1 h before the substrate was added to initiate the reaction; CA (1 h, 30) represents immobilized cells on the calcium alginate were incubated at 30 °C for 1 h before the substrate was added to initiate the reaction.

The thermal stability of cells of *G. candidum* AS 2.361 is one of the most important application criteria for different applications. Thus, the thermostability of *G. candidum* AS 2.361 cells was evaluated as well. Generally, the immobilized cells are superior to the free cells with respect to thermostability [39]. Thus, the thermostability of the immobilized cells on agar and calcium alginate, and the free cells were all characterized. All tested cells including immobilized cells and free cells were first incubated at various temperatures (30–70 °C) for 1, 3, 6, 9, and 12 h, respectively, prior to the addition to the reaction mixture. Then, the cells (3 g free cells or 4.5 g immobilized cells) were resuspended to 10 mL Na_2_HPO_4_-KH_2_PO_4_ buffer (100 mM, pH 7.0), containing 0.5 g glucose and 10 mM substrate. The reaction mixture was shaken at 25 °C for 24 h. As shown in Figure 4, the immobilized cells of *G. candidum* 2.361 on both agar and calcium alginate were stable at 30 °C or 40 °C for 12 h, without any activities loss (the activity of the enzyme without incubation at the given temperature was defined as the original activity and corresponds to 99% yield of the reduction product). Notably, the immobilized cells on calcium alginate kept >99% of their original activity after incubation at 50 °C for 1 h. When the temperature increased to 60 °C, a slight decrease in the activity (loss of 15% of the initial activity) was detected for the immobilized cells on calcium alginate. Remarkably, the immobilized cells on agar still showed a retention of >99% of their original activity after incubation at 60 °C for 1 h. After 3 h of incubation at 60 °C, the immobilized cells on agar lost 49% of the initial activity, and the immobilized cells on calcium alginate lost 57% of the initial activity, but both lost their activity almost completely after incubation at 70 °C for 1 h. On the other hand, the results showed that the free cells were stable at 30 °C for 1 h, without any activity loss, but started to lose the activity significantly when the temperature increased to 40, 50, and 60 °C. After incubation at 60 °C for 1 h, almost no activity was detected for the free cells. From the above results obtained, it is clear that the activity and stability of the immobilized cells are more resistant against heat than that of free cells. In the case of free cells and immobilized cells of strain *G. candidum*, AS 2.361 derivatives such effect of temperature on changes in activity and stability can be related to the protein unfolding and thus to the enzyme denaturation. The results also indicated that the profile curves for both immobilizations on agar and calcium alginate shifted towards higher temperatures suggesting that between enzyme and the tested supports there is existing a strong interaction, enhancing the conformation stability of the free cells.

#### 2.3.3. Storage Stability

**Figure 5:**
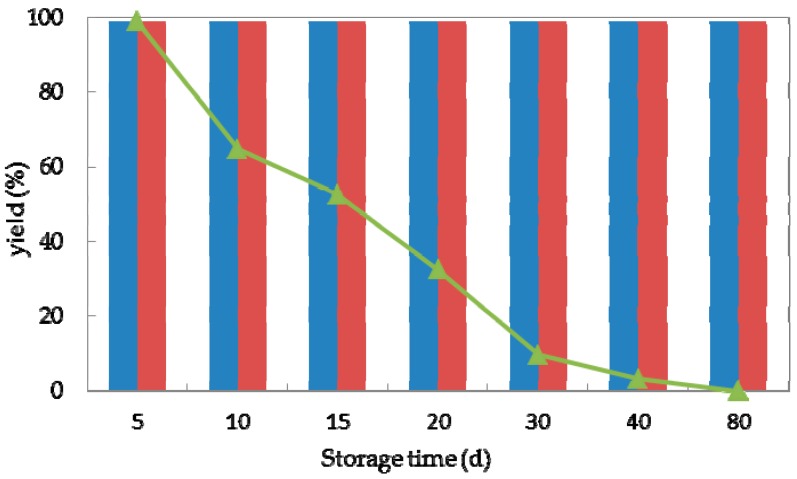
Storage stability of free cells (represented by the triangles), immobilized cells on agar (represented by the red striped bar) and calcium alginate (represented by the blue striped bar) of *G. candidum* AS 2.361 catalyzing the reduction of 1-(2-bromophenyl)ethanone (**1a**) at 4 °C.

Storage stability is an essential factor for the practical application of (bio)catalysts [40]. To investigate the storage stability of strain *G. candidum* AS 2.361, both immobilized cells and free cells were stored in physiological saline (0.85% NaCl, *w/v*) at 4 °C and tested for the activity in bioreduction of ketone **1a**. Samples (3 g free cells and 4.5 g immobilized cells) were withdrawn at various time intervals (5, 10, 20, 30, 40, and 80 days) and added to 10 mL Na_2_HPO_4_-KH_2_PO_4_ buffer (100 mM, pH 7.0), containing 0.5 g glucose and 10 mM substrate to initiate the reaction. The reaction of the mixture was then shaken at 25 °C for 24 h. As shown in Figure 5, the storability of the immobilized cells on the agar or calcium alginate was much superior to the free cells. The immobilized cells were stable at 4 °C for 80 days without any activity loss. The free cells could maintain 99% of their original activity after storage for 5 days at 4 °C. Decreases of 35, 48, and 68% of the initial activity were observed after storage at 4 °C for 10, 15, and 20 days, respectively. The free cells lost their activity completely after storage at 4 °C for 40 days. Hence, we concluded that after immobilization on agar or calcium alginate, strain *G. candidum* 2.361 exhibited excellent storage properties, which is essential for its industrial application potential. It needs to emphasize that in the current study of storage stability; all the cells were stored in physiological saline (0.85% NaCl, *w*/*v*) at 4 °C and were withdrawn at regular intervals for activity test. Effect of other factors such as storage solvent and storage temperature might cause the loss of activity and stability of cells of *G. candidum* AS 2.361, which should be considered for future studies.

#### 2.3.4. Reusability

**Figure 6:**
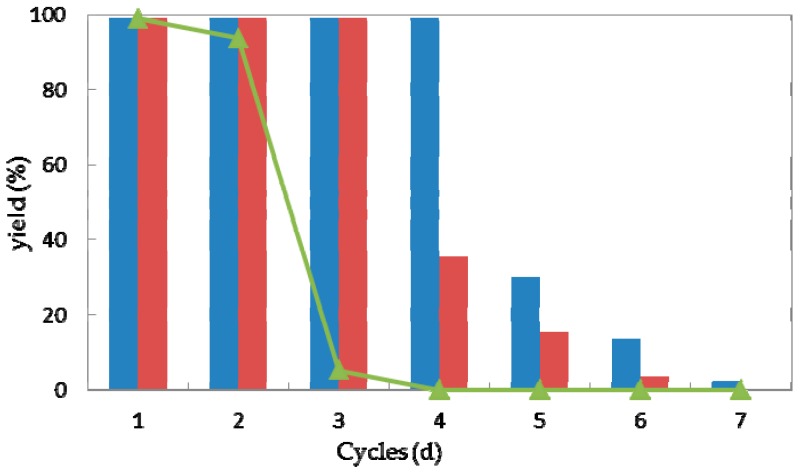
Repeated bioreduction of 1-(2-bromophenyl)ethanone (**1a**) catalyzed by free (triangles), agar-immobilized (blue striped bar) and calcium alginate-immobilized (red striped bar) cells of *G. candidum* 2.361 cells.

Reusability is one of the most important characteristics of a (bio)catalyst for practical application [41]. In the view of process economics, the higher the number of cycles that a (bio)catalyst remains stable, the more efficiently a process can be run. Taking the current high costs of enzymes, their features with the possibility of regenerating and reusing would be highly attractive. Thus high operational stability would improve the values of a biocatalyst [42,43,44]. To explore the operational stability of strain *G. candidum* AS 2.361, reactions were performed to examine the recyclability of both the immobilized cells and free cells. Every reaction was performed in 50-mL screw-capped plastic vials to prevent evaporation of ketones/alcohols. The reaction mixture contained 3 g of free resting cells or 4.5 g of immobilized cells, 0.5 g of glucose in 10 mL Na_2_HPO_4_-KH_2_PO_4_ buffer (100 mM, pH 7.0), with the substrate **1a** of the final concentration of 10 mM. The solution was shaken at 25 °C for 24 h. After the completion of the reaction, the cells were separated by centrifugation (4000 rpm, 20 min, 4 °C), washed twice with the same buffer (Na_2_HPO_4_-KH_2_PO_4_ buffer (100 mM, pH 7.0)) and then reused in the next cycle under the same reaction conditions. As shown in Figure 6, the immobilized cells on the agar and calcium alginate showed superior recyclability compared to free cells during successive cycles of catalytic asymmetric bioreduction of aromatic ketone **1a**. The immobilized cells on agar showed high activity and complete conversion of substrate **1a** for four cycles. Loss of 70% of initial activity was detected at the fifth cycle and almost complete loss of the activity was detected on the seventh cycle. The immobilized cells on calcium alginate showed high activity and enantioselectivity towards substrate **1a**, without any loss of activity or enantioselectivity for three cycles. Losses of 64% and 85% and of the initial activity were observed for cycles four and five, whereas almost no activity (3.7% yield of desired product) was retained inthe seventh cycle. The free cells could maintain approximately 92% of their original activity during the second cycle but were almost deactivated in the third cycle. Notably, the recycling of the immobilized or free cells showed no significant impact on the enantioselectivities of the biocatalysts, as supported by the observation that the product *ee* remained consistently higher than 99.0% during each cycle of the reaction (data not shown). The results demonstrate the high repeated use by the immobilized cells, suggesting that the immobilized *G. candidum* AS 2.361 on agar or calcium alginate would be promising for application in actual industrial production. Biocatalysts (cells of *G. candidum* AS 2.361) inactivation in this specific case of changes in activity and stability becomes one of the greater problems when they are used as industrial catalysts. Inactivation of a cell starts by some reversible conformational changes, and finally the cell may also suffer some chemical modifications, aggregation, etc. That way, most strategies to stabilize cells are directed to the slowdown of these initial conformational changes. The conformational changes start by some weak point of the cell conformation and then get more generalized along the whole cell’s conformation until reaching full cell inactivation. However, it is not difficult to imagine that the weakest point of a cells conformation, or at least the way that the cells structure follows during inactivation, may be different under different inactivating conditions [45].

## 3. Materials and Methods

### 3.1. General

Chemicals used in the current study were all purchased from Sigma-Aldrich (Schnelldorf, Denmark) and were used directly without any purification. *G. candidum* AS 2.361 culture media components were purchased from Huankai Microbial (Zhuhai, China).

NMR data were obtained with a Bruker Advance 500 instrument (^1^H 500 and ^13^C 125 MHz, respectively, (Beijing Topnovo technology Co., Ltd, Beijing, China) and internally referenced to residual solvent signals. Data for ^1^H NMR are recorded in terms of chemical shift (d ppm), multiplicity (s = singlet, d = doublet, t = triplet, q = quartet, m = multiplet), integration, coupling constant (Hz), and assignment. Data for ^13^C NMR are recorded as chemical shift. Optical rotations were measured at 20 °C on an MCP 300 (Anton Paar, Vienna, Austria) (sodium D line). Column chromatography was performed with a silica gel (0.060–0.200 mm, pore diameter ca. 6 nm), using petroleum ether (PE) and ethyl acetate (EtOAc) as eluents. Thin-layer chromatography (TLC) was performed on 0.20 mm silica gel 60-F plates. Organic solvents were removed using a rotary evaporator. Free cells of *G. candidum* AS 2.361 were obtained as described in a previous report by our group [19]. *Geotrichum candidum* AS 2.361 used in this study was deposited and commercially available in Guangdong Culture Collection Center (http://www.gimcc.net/index.asp), which belongs to the World Federation for Culture Collections (Budapest Notification no. 309).

### 3.2. Immobilization of G. candidum AS 2.361

#### 3.2.1. Agar Immobilization

5 g of free resting cells were resuspended to 4 mL distilled water (1.25 g/mL). The resuspension was added to a 50-mL screw-capped glass vial containing 20 mL sterilized agar (5% *w*/*v*). After stirring thoroughly for 5 min, the mixture was transferred into a clean plate. The solution was allowed to solidify, forming a solid agar layer. The agar layer was cut into small pieces (3 × 3 × 3 mm^3^), and then stored in a phosphate buffer (pH 7, 0.1 M) at 4 °C after washing with distilled water.

#### 3.2.2. Calcium Alginate Immobilization

5 g of free resting cells were resuspended to 10 mL distilled water (0.2 g/mL). The resuspension was added to a 50-mL screw-capped glass vial containing 5 mL sterilized sodium alginate (40 g/L) solution. After stirring for 5 min, the mixture was added dropwise into a sterilized solution of CaCl_2_ (4% *v*/*v*). The formed stable bead layers were kept in a 20–22 °C water bath for 2 h. The collected spherical particles was stored in CaCl_2_ (4%) solution at 4 °C after washing thoroughly with distilled water.

### 3.3. SEM Analysis

After washing with water to remove the non-adhering support matrix, the immobilized cells were successively suspended in ethanol of different concentrations (10, 30, 50, 70, 90, and 100%) for 15 min. The samples were air dried at room temperature in between. Using a Baltec MCS 010 model sputter (Verschleiss Schutz Technik Keller GmbH & Co KG, Balzers, Liechtenstein), the washed immobilized cells were coated with 8–10 nm of gold by argon ion sputtering, before used for scanning electron microscopy on a Joel JMS 6480 LV computer (TECHCOMP(CHINA)LTD., Guangzhou, China).

### 3.4. Reduction of Ketones

Reactions were all performed in 50-mL screw-capped plastic vials to prevent evaporation of ketones/alcohols. The reaction mixture contained 3 g of free resting cells (0.3 g/mL) or 4.5 g of immobilized cells (0.45 g/mL), 0.5 g of glucose in 10 mL Na_2_HPO_4_-KH_2_PO_4_ buffer (100 mM, pH 7.0), with the substrates of the final concentration of 10 mM. The solution was shaken at 25 °C for 24 h. After separating from the cells by centrifugation, 2 mL of the obtained supernatant was saturated with NaCl, and extracted with *n*-hexane/*i*-PrOH (95/5, *v*/*v*, 2 × 1 mL). The obtained organic layer was dried over Na_2_SO_4_ and injected into HPLC for yield and *ee* measurement. The yield and enantioselectivity excess (*ee*) were recored on a chiral HPLC using a Shimadzu LC-10AT VP series and a Shimadzu SPD-M10Avp photo diode array detector (190–370 nm) with a Chiralcel AD-H column (eluent: *n*-hexane/*i*-PrOH (95:5, *v/v*), flow rate: 0.5 mL/min, column temperature 25 °C). The retention time of substrates and desired products are summarized as below: 11.85 min for **1a**, 12.71 min for (*R*)-**2a**, 13.23 min for (*S*)-**2a**; 10.66 min for **1b**, 16.45 min for (*R*)-**2b**, 17.39 min for (*S*)-**2b**; 11.21 min for **1c**, 16.83 min for (*R*)-**2c**, 17.99 min for (*S*)-**2c**; 10.71 min for **1d**, 13.32 min for (*R*)-**2d**, 13.98 min for (*S*)-**2d**; 10.49 min for **1e**, 14.60 min for (*R*)-**2e**, 16.35 min for (*S*)-**2e**; 10.78 min for **1f**, 15.09 min for (*R*)-**2f**, 16.09 min for (*S*)-**2f**; 11.46 min for **1g**, 15.93 min for (*R*)-**2g**, 17.93 min for (*S*)-**2g**; 10.94 min for **1h**, 14.74 min for (*R*)-**2h**, 16.49 min for (*S*)-**2h**; 16.75 min for **1i**, 20.98 min for (*R*)-**2k**, 24.71 min for (*S*)-**2i**; 16.42 min for **1j**, 22.33 min for (*R*)-**2j**, 27.39 min for (*S*)-**2j**.

The absolute configuration of products was assigned by measuring and comparing the optical rotation of isolated alcohols and those reported in previous studies [33,34,35,36]: (*S*)-**2a**, [α${]}_{D}^{20}$ = −62.4 (*c* 1.00, CHCl_3_), {lit.[33] (*S*)-1-(2-bromophenyl)ethanol [α${]}_{D}^{25}$ = −28.8 (*c* 1.00, CHCl_3_)}; (*S*)-**2b**, [α${]}_{D}^{20}$ = −43.9 (*c* 1.00, CHCl_3_), {lit.[33] (*S*)-1-(3-bromophenyl)ethanol [α${]}_{D}^{25}$ = −27.6 (*c* 1.00, CHCl_3_)}; (*S*)-**2c**, [α${]}_{D}^{20}$ = −17.3 (*c* 1.00, MeOH), {lit.[34] (*S*)-1-(4-bromophenyl)ethanol [α${]}_{D}^{21}$ = −20.6 (*c* 1.07, MeOH)}; (*S*)-**2d**, [α${]}_{D}^{20}$ = –78.4 (*c* 1.00 in MeOH), {lit.[35] (*S*)-1-(2-chlorophenyl)ethanone [α${]}_{D}^{26}$ = −42.5 (*c* 0.85 CHCl_3_)}; (*S*)-**2e**, [α${]}_{D}^{20}$ = –49.7 (*c* 1.00 in MeOH), {lit.[33] (*R*)-1-(3-chlorophenyl)ethanone [α${]}_{D}^{25}$ = −36.7 ο (*c* 0.71, CHCl_3_)}; (*S*)-**2f**, [α${]}_{D}^{20}$ = –66.49 (*c* 1.00 in MeOH), {lit.[35] (*R*)-1-(4-chlorophenyl)ethanone [α${]}_{D}^{26}$ = −22 (*c* 0.53 in Et_2_O)}; (*S*)-**2h**, [α${]}_{D}^{20}$ = –38.5 (*c* 1.00 in MeOH), {lit.[35] propiophenone [α${]}_{D}^{26}$ = −40 (*c* 0.85, CHCl_3_)}; (*S*)-**2i**, [α${]}_{D}^{20}$ = + 9.68 (*c* 0.05 in MeOH), {lit.[36] (*R*)-2-chloro-1-phenylethanone [α${]}_{D}^{20}$ = −50.5 (*c* 1.00, CHCl_3_)}; (*R*)-**2j**, [α${]}_{D}^{20}$ = –16.9 (*c* 0.35 in MeOH), {lit.[36] (*R*)-2-bromo-1-phenylethanone [α${]}_{D}^{20}$ = –30.9 (*c* 1.00 in CHCl_3_)}.

### 3.5. Preparative-Scale Synthesis of (S)-1-(2-bromophenyl) ethan-1-ol (S-**2a**)

1-(2-bromophenyl) ethanone (**1a**, 1.98 g, 10 mmol) was dissolved in 1000 mL of Na_2_HPO_4_-KH_2_PO_4_ buffer (100 mM, pH 7.0) containing 50 g glucose and 450 g immobilzed cells of G. candidum AS 2.361 on agar. The reaction mixture was shaken at 25 °C with the speed of 220 rpm for 24 h. The cells were separated by centrifugation and the obtained supernatant (about 1000 mL) was saturated with NaCl and then extracted with ethyl acetate (2 × 1000 mL). The combined organic layers were dried with Na_2_SO_4_ and evaporated under reduced pressure. The crude product mixture was purified by flash chromatography on silica gel (ethyl acetate/petroleum ether, 1:5) to yield 1.90 g (95%) of a colourless oil (*S*-**2a**). The NMR data of isolated (*S*)-**2a** was in accordance with literature [33].

### 3.6. pH Tolerance and Thermostability

Reactions were all performed in 50 mL screw-capped plastic vials to prevent evaporation of ketones/alcohols. The reaction mixture contained 3 g of free resting cells (0.3 g/mL) or 4.5 g of immobilized cells (0.45 g/mL), 0.5 g of glucose in 10 mL given buffer (100 mM) with given given pH values between 3.5–11 (citrate buffer of pH 3.5 and 4.5; phosphate buffer of pH 5.5, 6.5 and 7.5; Tris buffer of pH 8.5, 9.0, 10.0, and 11.0), with the substrates of the final concentration of 10 mM. The solution was shaken at 25 °C for 24 h. After separating from the cells by centrifugation, 2 mL of the obtained supernatant was saturated with NaCl, and extracted with *n*-hexane/*i*-PrOH (95/5, *v*/*v*, 2 × 1 mL). The obtained organic layer was dried over Na_2_SO_4_ and injected into HPLC for yield and *ee* measurement, as described above in Section 3.4.

All tested cells including immobilized cells and free cells were first incubated at various temperatures (30–70 °C) for 1, 3, 6, 9, and 12 h, respectively, prior to the addition to the reaction mixture. Then, the cells (3 g free cells or 4.5 g immobilized cells) were resuspended to 10 mL Na_2_HPO_4_-KH_2_PO_4_ buffer (100 mM, pH 7.0), containing 0.5 g glucose and 10 mM substrate. The reaction mixture was shaken at 25 °C for 24 h. The yields and enantioselectivity excess (*ee*) were obtained by a chiral HPLC, as described above in Section 3.4.

### 3.7. Storage Stability and Reusability

Both immobilized cells and free cells were stored in physiological saline (0.85% NaCl, *w*/*v*) at 4 °C and tested for the activity in bioreduction of ketone **1a**. Samples (3 g free cells (0.3 g/mL) and 4.5 g immobilized cells (0.45 g/mL)) were withdrawn at various time intervals (5, 10, 20, 30, 40, and 80 days) and added to 10 mL Na_2_HPO_4_-KH_2_PO_4_ buffer (100 mM, pH 7.0), containing 0.5 g glucose and 10 mM substrate to initiate the reaction. The reaction of the mixture was then shaken at 25 °C for 24 h. The yields and enantioselectivity excess (*ee*) were obtained using a chiral HPLC, as described above in Section 3.4.

Every reaction was performed in 50 mL screw-capped plastic vials to prevent evaporation of ketones/alcohols. The reaction mixture contained 3 g of free resting cells (0.3 g/mL) or 4.5 g of immobilized cells (0.45 g/mL), 0.5 g of glucose in 10 mL Na_2_HPO_4_-KH_2_PO_4_ buffer (100 mM, pH 7.0), with the substrate **1a** of the final concentration of 10 mM. The solution was shaken at 25 °C for 24 h. After the completion of the reaction, the cells were separated by centrifugation (4000 rpm, 20 min, 4 °C), washed twice with the same buffer (Na_2_HPO_4_-KH_2_PO_4_ buffer (100 mM, pH 7.0)) and then reused in the next cycle under the same reaction conditions. 2 mL of the obtained supernatant was saturated with NaCl, and extracted with *n*-hexane/*i*-PrOH (95/5, *v*/*v*, 2 × 1 mL). The obtained organic layer was dried over Na_2_SO_4_ and injected into HPLC for yield and *ee* measurement, as described above in Section 3.4.

## 4. Conclusions

In this study, we reported the activity, stability (pH tolerance, thermostability, and storage stability) and reusability of a marine-derived fungus *G. candidum* AS 2.361. To achieve this goal, two differently immobilized cells (agar-immobilized cells, calcium alginate-immobilized cells) and free cells were prepared for the asymmetric reduction of 14 aromatic ketones. The immobilization was characterized by FTIR and SEM, revealing that the cells were successfully entrapped on agar and calcium alginate. pH tolerance studies revealed that the immobilized cells gave 99% yield of the product (*ee* > 99%) at pH values in the range of 3–9, whereas the free cells exhibited an optimal activity at pH 7.0, yielding 99% of the desired product with >99% *ee* but were found to be very sensitive to alkaline and acidic conditions. Thermostabilities studies showed that immobilized cells on agar maintained 99% of the original activity during 1 h of incubation at 60 °C or 12 h of incubation at 40 °C. The immobilized cells maintained 99% of the original activity and enantioselectivity after storage at 4 °C for 80 days. Batch experiments showed that the immobilized cells on agar could be reused for four cycles without significant loss of activity and enantioselectivity. In summary, immobilized *G. candidum* AS 2.361 on agar or calcium alginate showed better acid–alkaline resistance, higher thermostability, better storage stability, and reusability as well as lower production costs compared to free cells. These results also indicated that immobilized *G. candidum* AS 2.361 can provide a solution for practical application and is a useful choice for the preparation of chiral alcohols.

## Figures and Tables

**Table 1:**
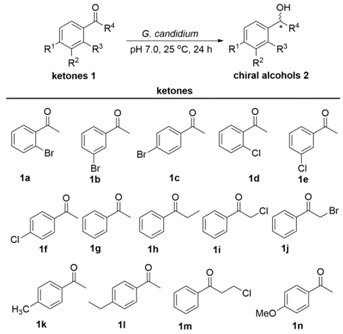
Comparison of biocatalytic activity of free and immobilized cells for asymmetric reduction of various aromatic ketones ^a^.

Substrate	Free Cells		Immobilized Cells on Agar		Immobilized Cells on Calcium Alginate	
	Yield (%)	*ee* (%)	Yield (%)	*ee* (%)	Yield (%)	*ee* (%)
1a	99	99 (*S*)	99	99 (*S*)	99	99 (*S*)
1b	44	58 (*S*)	65	99 (*S*)	39	74 (*S*)
1c	49	75 (*S*)	37	8 (*S*)	45	32 (*S*)
1d	81	90 (*S*)	96.7	99 (*S*)	33	99 (*S*)
1e	96	99 (*S*)	66	99 (*S*)	89	91 (*S*)
1f	54	59 (*S*)	53	32 (*S*)	35	19 (*S*)
1g	63	87 (*S*)	86	99 (*S*)	28	99 (*S*)
1h	52	81 (*S*)	36	97 (*S*)	48	99 (*S*)
1i	24	18 (*S*)	41.5	45 (*R*)	39	38 (*R*)
1j	41	87 (*R*)	34.3	99 (*R*)	33	69 (*R*)
1k	n.d.	n.d.	n.d.	n.d.	n.d.	n.d.
1l	n.d.	n.d.	n.d.	n.d.	n.d.	n.d.
1m	n.d.	n.d.	n.d.	n.d.	n.d.	n.d.
1n	n.d.	n.d.	n.d.	n.d.	n.d.	n.d.

^a^ Reaction conditions: 10 mL Na_2_HPO_4_-KH_2_PO_4_ buffer (100 mM, pH 7.0), 3 g resting cells (or 4.5 immobilized cells), 10 mM substrate, 0.5 g glucose, 25 °C, 24 h; Yield and *ee* were determined by chiral HPLC analysis equipped with a Chiracel AD-H chiral column (see Experimental Section 3.1); The absolute configuration was assigned by comparing the specific signs of rotation measured for the isolated products with those reported in the literature [33,34,35,36].
